# Role of Raf-kinase inhibitor protein in colorectal cancer and its regulation by hydroxycamptothecine

**DOI:** 10.1186/s12929-015-0162-y

**Published:** 2015-07-16

**Authors:** Fang Nie, Jianguo Cao, Jinlu Tong, Mingming Zhu, Yuan Gao, Zhihua Ran

**Affiliations:** Department of Intensive Care Medicine, Ren Ji Hospital, School of Medicine, Shanghai Jiao Tong University, Shanghai, China; Division of Gastroenterology and Hepatology, Ren Ji Hospital, School of Medicine, Shanghai Jiao Tong University, Shanghai Institute of Digestive Disease, 145 Middle Shandong Road, Shanghai, 200001 China

**Keywords:** Colorectal cancer, Hydroxycamptothecine, Role, Raf-kinase inhibitor protein

## Abstract

**Background:**

Recently accumulated evidence suggests that Raf kinase inhibitor protein (RKIP) participates in regulation of many signaling pathways and plays an important role in tumorigenesis and tumor metastasis. However, studies investigating the role of RKIP in colorectal cancer have not been reported. The aim of this study was to investigate the role of RKIP on colorectal cancer cell differentiation, progression and its correlation with chemosensitivity.

**Results:**

Immunohistochemical analysis revealed that RKIP expression was higher in non-neoplastic colorectal tissue (NCRCT) and colorectal cancer tissue (CRCT) than that in metastatic lymph node tissue (MLNT) (*P* <0.05). P-ERK protein expression was higher in MLNT and CRCT than that in NCRCT (*P* = 0.02). Immunocytochemical analysis further revealed that RKIP expression was higher in the well differentiated cell line SW1116 as compared to that in the poorly differentiated cell line LoVo. Matrigel invasive assay demonstrated that the inhibition of RKIP by short hairpin RNA (shRNA) 271 transfection significantly increased the number of migrated cells (90.67 ± 4.04 *vs*. 37.33 ± 2.51, *P* <0.05), whereas over-expression of RKIP by PEBP-1 plasmid transfection significantly suppressed the number of migrated cells (79.24 ± 5.18 *vs*. 154.33 ± 7.25, *P* <0.05). Meanwhile, down-regulation of RKIP induced an increase in the cell survival rate by inhibiting apoptosis induced by hydroxycamptothecine.

**Conclusions:**

RKIP was also found to be associated with cell differentiation, with a higher activity in well differentiated colorectal cancer cells than in poorly differentiated ones. The upregulated expression of RKIP in colorectal cancer cells inhibited cell invasion and metastasis, while downregulation of RKIP reduced chemosensitivity by inhibiting apoptosis induced by HCPT.

## Background

The Raf kinase inhibitor protein (RKIP), a member of an evolutionarily conserved family of phosphatidylethanolamine-binding proteins (PEBPs), has been recognized as a representative of a new class of modulators of signaling cascades that serve to maintain the balance of biological systems. RKIP inhibits kinase signaling pathways and metastasis. A vast array of studies have been conducted to understand the role of RKIP in various malignancies such as melanoma [1], prostate cancer [2], breast cancer [3], gastric cancer [4], glioblastoma [5], lung cancer [6], and myelogenous leukemia [7]. Most notably, RKIP has been identified as a modulator of extracellular signal-regulated kinase (ERK) [8], nuclear factor-kappa B (NFkB) [9], and G protein coupled receptor (GPCR) [10, 11] signaling cascades. Moreover, RKIP has been implicated in tumor biology. Down-regulation of RKIP significantly correlated with radio-resistance-induced relapse [12]. RKIP sensitizes prostate and breast cancer cells to drug-induced apoptosis [13]. RKIP as a suppressor of metastasis has been confirmed as a predictor of cancer progression and is even being considered as a potential therapeutic target.

Recently, RKIP was found to inhibit proliferation of activated hepatic stellate cells by down-regulation of mitogen-activated protein kinase (MAPK) signaling, but was found to promote the migration of hepatic stellate cells and wound closure [14]. Surprisingly, in the merkel cell carcinoma cell line UISO, RKIP knockdown does not alter proliferation or chemosensitivity to cisplatin, and has no effect on blocking the MAP kinase pathway [15]. RKIP does not appear to play exactly the same role in different cancers and non-malignant cells. This may be because of the diverse molecular functioning milieu, different cell proliferation signaling pathways active in different tissues and other mechanisms that are as yet unknown.

In developed countries, colorectal cancer (CRC) is the third-most frequent cancer and the second-most common cause of cancer-related death [16]. Chemotherapeutic drug resistance remains a major obstacle to the successful treatment of colorectal cancer. RKIP silencing has been correlated significantly with KEAP1 degradation and bestowed resistance to cisplatin by up-regulating NRF2 gene in HER-293 cells. Also, expression levels of RKIP/KEAP1 have relationship with the extent of apoptosis after treatment with adriamycin in HT29 cells [17]. In normal colonic mucosa, cytoplasmic RKIP expression was found in 100 % of cells [18]. A study suggested that RKIP is probably a downstream effector in TWEAK (Tumor necrosis factor-like weak inducer of apoptosis) mediated invasion inhibition. Knockdown of RKIP expression in HCT116 cells by short hairpin RNA (shRNA) resulted in increased invasiveness [19]. Loss of RKIP was also found to be associated with N stage disease, the presence of vascular invasion, metastasis and worse survival time in mismatch repair–proficient CRC [18]. RKIP expression in primary CRCs can be useful for identifying early-stage CRC patients at risk of relapse [20]. It is plausible that RKIP plays an important role in the occurrence and development of colorectal cancer. However, few studies exploring the role of RKIP in colorectal cancer have been reported and the specific mechanism still remains unresolved.

In our paper, we shall first investigate the effect of RKIP on differentiation, invasion and metastasis of colorectal cancer cells, both *in vivo* and *in vitro*. Later, the association between RKIP and chemosensitivity to hydroxycamptothecine (HCPT) would be assessed, including the underlying mechanism.

## Methods

### Cell culture

Two human colorectal cancer cell lines SW1116 and SW480 were cultured in RPMI 1640 medium (Gibco, Carlsbad, CA) with 10 % fetal bovine serum. LoVo cells (human colon adenocarcinoma cell line) were cultured in F-12 k medium with 10 % fetal bovine serum. Two other human colorectal cancer lines, HT-29 and HCT-116 were cultured in McCoy’s 5A medium (Sigma, Louis, MO) with 10 % fetal bovine serum. Caco-2 (human colon adenocarcinoma cell line) cells were cultured in DMEM medium (Gibco, Carlsbad, CA) with 10 % fetal bovine serum. All cells were incubated at 37 °C in humidified 5 % CO_2_. HCPT-resistant cell line of human colon cancer cell, SW1116/HCPT, was developed by stepwise increasing concentration method [21].

### Patients and tissue samples

Thirty non-neoplastic colorectal tissue (NCRCT) samples and 30 colorectal cancer tissue (CRCT) samples were surgically obtained from patients admitted from 2007 to 2009 in the Department of General Surgery at Shanghai Renji Hospital, China. Another 30 matching metastatic lymph node tissue (MLNT) samples archived in formalin-fixed paraffin-embedded tissue blocks were obtained from Department of Pathology, Shanghai Renji Hospital, China.

### Immunohistochemistry and immunocytochemistry

For immunohistochemistry (IHC), sections of paraffin-embedded tissues were deparaffinized in xylene, rehydrated in graded alcohol solutions, and treated with an antigen retrieval solution (10 mmol/L sodium citrate buffer, pH 6.0). For IHC, cells were incubated at the density of 2 × 10^4^ cells/cm^2^ in 6-well culture plate with glass slice. A three-step streptavidin-biotin horseradish peroxidase method was used, and the expression of RKIP and pERK was examined with the primary antibodies (RKIP, Santa Cruz, dilution 1:200; pERK, Santa Cruz, dilution 1:50) using the SP kit (ZYMED, San Diego, America), according to the manufacturer’s instructions. Protein expression was quantified based on a 3-point positive scale (+: 0 to 2; ++: 3 to 4; +++: 5 to 9). The scale ranking equaled the product of staining intensity score (4-point scale: negative-0; weak-1; intermediate-2; and strong-3) and staining extent score (percentage of positive tumor cells: 0–0 % to 10 %; 1–15 % to 50 %; 2–51 % to 75 % and 3–>75 %). The percentage of positive cells was calculated by counting more than 100 cancer cells in randomly selected high-power fields (400×). All samples were analyzed by two senior pathologists.

### Real-time polymerase chain reaction

One μg of RNA was reverse transcribed using RT kit (Takara, Tokyo, Japan). Quantitative real-time polymerase chain reaction (PCR) was performed with a SYBR Green real-time premix kit (Takara, Tokyo, Japan). Forward (F) and reverse (R) primer sequences were as follows: RKIP(F)5′-agcagtggcacagtcctc-3′,(R)5′-tggtctccagatcggttg-3′; GAPDH(F)5′-gcaccgtcaaggctgagaac-3′, (R) 5′-atggtggtgaagacgccagt-3′. PCR was performed for 30s at 95 °C, 5 s at 95 °C and 31 s at 60 °C for 40 cycles.

### Stable transfection and G418 screening of stable expression cell lines

PGPU6/GFP/Neo-PEBP1-501/271/376 (RKIP shRNA vector, RKIP GenBank Number: NM-002576) and PGPU6/GFP/Neo-sh NC (negative control) were obtained from Gene Pharma, Inc. (Shanghai, Co). SW1116 cells were transfected with each shRNA using Lipofectamine 2000 reagent (Invitrogen, Carlsbad, CA) as per the manufacturer’s protocol. The silencing effect of RKIP was confirmed by real-time PCR and Western blot analysis. SW1116 cells were transfected with the shRNA that had the best silencing effect, and screened by G418 (Sigma). The best screening concentration of G418 for SW1116 cells was 800 μg/mL. After one month of selection in RIPM-1640 medium containing 800 mg/mL G418. Individual neomycin-resistant colonies were isolated and expanded.

### Western blotting

Western blot analysis was performed using the standard methodology as described previously [22]. The cells were lysed in RIPA Lysis Buffer (Beyotime, Shang-hai, CH); equal amount of protein (100 μg/lane) from whole-cell lysates were subjected to SDS-PAGE. Proteins were transferred to microporous PVDF membranes (Millipore, Massachusetts, CA) and were probed with specific primary antibodies, followed by the appropriate HRP-conjugated secondary antibodies (Pierce, Rockford, USA). The antibodies used in this study were rabbit monoclonal antibody RKIP (Santa Cruz, Inc, 1:200 dilution), rabbit polyclonal antibody P-ERK (Santa Cruz, Inc, 1:100 dilution), GAPDH (Kang-chen, Shang-hai, CH. 1:1000). Proteins were detected using the enhanced chemiluminescence detection kit (Super Signal West Femto Substrate, Pierce).

### Apoptosis assay

Apoptosis was demonstrated using Annexin V-FITC apoptosis detection kit (Abcam, USA) according to manufacturer’s protocol. About 1 × 10^6^ cells were collected and immediately analysed using a flow cytometer (BD, San Diego, CA).

### Matrigel invasion assay

Matrigel invasion assay was performed using transwell insert chamber (8.0 μm pore polycarbonate membranes, Corning, Inc.) coated with Matrigel (BD, USA) as per the manufacturer’s protocol. Then 1 × 10^5^ transfected cells were harvested and seeded in 0.1 % FBS medium into the upper chamber, whereas medium supplemented with 20 % FBS was applied to the lower chamber as a chemo attractant for inducing invasion. After 48 h of incubation, the migrant cells which were attached to the lower surface were fixed in 4 % paraformaldehyde and stained with crystal violet. The number of migrated cells on the lower surface of the membrane was counted under an invert microscope by examining 10 fields.

### MTT assay and flow cytometry

Cell viability was measured using MTT (3-(4,5-dimethylthiazol-2-yl)-2-5 diphenyl tetrazolium bromide) assay. 5 × 10^3^ cells were incubated in a 96-well culture plate for 24 h followed by their treatment with different concentrations of HCPT for 48 h. The supernatant was removed and 180 μL cell culture fluid was added. 10 μL of MTT (Sigma, M5665) dissolved in 100 μL of phosphate-buffered saline (Sigma P4417) was added followed by incubation for 4 h at 37 °C in a humid, 5 % CO_2_ atmosphere. After this, the supernatant was removed, and the insoluble formazan crystals were dissolved in 150 μL of dimethyl sulfoxide. The absorbance was read in a microplate reader model 550 ELISA plate reader (BioRAD, Hercules, USA) at 570 nm.

### Statistical analysis

All statistical analyses were performed with SPSS version 13.0 software (SPSS, Chicago, IL, USA). The results of IHC were analyzed using Pearson’s chi-square-test and descriptive statistics. In-vitro results were expressed as mean ± standard deviation, and the data were analyzed for significance by ANOVA. A value of *P* <0.05 was considered to be statistically significant.

## Results

### Detection of RKIP and P-ERK expression in CRCT, NCRCT and MLNT by immunostaining

**Fig. 1:**
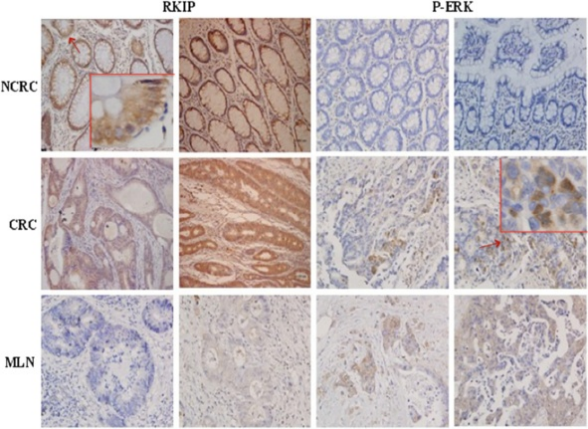
Immunohistochemical staining of NCRCT, CRCT and MLNT with anti-RKIP and anti-P-ERK antibody. Strong RKIP expression was stained in NCRCT, CRCT; and weak in MLNT In contrast, P-ERK was weak to moderate in CRCT, MLNT, and no staining was observed in NCRCT. *Magnification x100; Red arrows indicate the enlarged areas in red frames. Abbreviations: RKIP, Raf kinase inhibitor protein; NCRCT, non neoplastic colorectal tissue; CRCT, colorectal cancer tissue; MLNT, metastatic lymph node tissue*

**Fig. 2:**
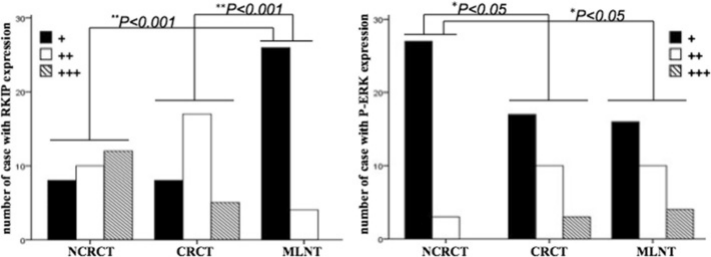
The variable expression of RKIP protein and P-ERK protein in different tissues by clustered charts. X-axis represented a different tissue. NCRCT: (*N* = 30); CRCT: (*N* = 30); MLNT: (*N* = 30); The positive staining of RKIP and P-ERK in different tissues are shown in Table 1. For RKIP: CRCT, NCRCT *vs*. MLNT (*x* ^2^ = 36.446 > × 20.05(4), **P <0.001). For P-ERK: MLNT, NCRCT *vs*. CRCT (*x* ^2^ = 11.675 > *x* ^2^ = 20.05(4), *P <0.05); *Abbreviations: RKIP, Raf kinase inhibitor protein; NCRCT, non neoplastic colorectal tissue; CRCT, colorectal cancer tissue; MLNT, metastatic lymph node tissue*

The expression of RKIP was found throughout in the cytoplasm of NCRCT and CRCT, with no significant difference between the two. However, the expression negligible in MLNT. The protein expression of P-ERK was positive in CRCT and MLNT, while it was negative in NCRCT. P-ERK staining was also mainly in the cytoplasm, with occasional nuclear staining (Fig. 1). The staining for RKIP was strong in NCRCT with “3+” and in CRCT with “2+”, while only very weak staining was detected in MLNT. The staining for P-ERK was very weak in NCRCT with no “3+”, while strong staining was there in NCRCT and CRCT. To summarize, RKIP expression levels were higher in CRCT and NCRCT than in MLNT (*χ*^2^ = 36.446, ^**^P <0.001). P-ERK expression levels were higher in MLNT and NCRCT than in CRCT (*χ*^2^ = 11.675, ^*^P <0.05) (Table 1 and Fig. 2).

**Table 1:** The staining intensity of RKIP and P-ERK

Colorectal tissue(N)	^**^RKIP			^*^P-ERK		
	+	++	+++	+	++	+++
NCRCT(30)	8	10	12	27	3	0
CRCT(30)	8	17	5	17	10	3
MLNT(30)	26	4	0	16	10	4

*NCRCT, non neoplastic colorectal tissue; CRCT, colorectal cancer tissue; MLNT, metastatic lymph node tissue;* Statistical analysis: One-way analysis of variance was followed by the student-newman-keuls post-hoc test. (**P <0.001, *P <0.05)

### Role of RKIP expression in colonic cancer differentiation

**Fig. 3:**
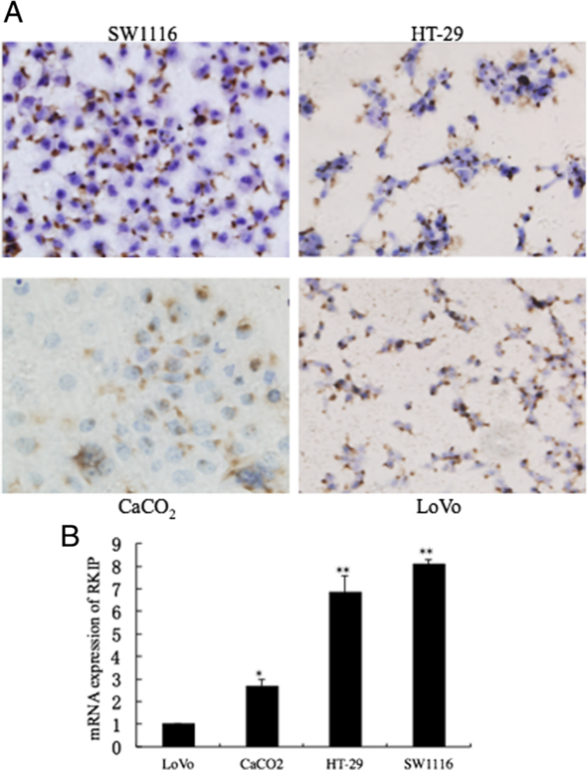
RKIP expression in colorectal cancer cell lines; **a** Immunohistochemical staining of colorectal cancer cell lines (LoVo, CaCO2, HT-29, SW1116) with an RKIP antibody. RKIP expression was present in an area of the cytoplasm. RKIP was strongly stained in SW1116 cells. Hematoxylin was used for nuclear staining (Magnification ×400); **b** mRNA expression of RKIP in cultured colorectal cancer cell lines. RKIP mRNA was detected by RT-PCR analysis, in 4 colorectal cancer cell lines (LoVo, CaCO2, HT-29, SW1116). *Abbreviations: RKIP, Raf kinase inhibitor protein; RT-PCR, reverse transcriptase- polymerase chain reaction*

We selected colon cell lines ranging from low-to-high grade of differentiation. RKIP was detected in some part of cytoplasm in all cell lines. The results showed that the expression of RKIP were higher in well differentiated cell lines (HT-29, SW1116) than in poorly differentiated cell lines (LoVo) (Fig. 3a). At the same time, RKIP mRNA expressions in these colon cancer cell lines were also illustrated by real-time PCR. RKIP expression was highest in SW1116 cell line and the lowest in LoVo cell line (Fig. 3b: ^*^P <0.05; ^**^P <0.01). These results show the consistency in the results attained by two different approaches. The expression of RKIP was correlated to the degree of tumor cell differentiation. The staining intensity of RKIP protein decreased with the reducing differentiation.

### The sub clone cells stably expressing RKIP antisense RNA were selected by G418 and named as SW1116/RKIP^—^. The eukaryotic expression vector PIRES_2_-EGFP/PEBP-1 can up-regulate RKIP expression in LoVo cells

**Fig. 4:**
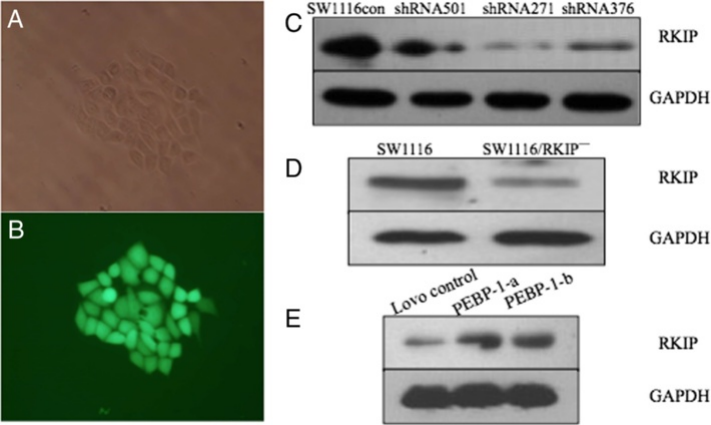
Establishment of the single clone SW1116/RKIP^—^ cells and effect of shRNA vector (PGPU6/GFP/Neo-PEBP1-501/271/376 targeted RKIP), over-expression vector (pIRES2-EGFP/PEBP-1). **a** SW1116 cells stably transfected with shRNA 271 were screened with G418 in 800ug/mL to obtain single colony selection in 24 well culture plates under optical microscope; **b** A under fluorescence microscope; **c** SW1116 cells were stably transfected with either empty shRNA vector PGPU6/ GFP/Neo (SW1116 con) or shRNA vector PGPU6/GFP/Neo-PEBP1 -501/271/376 target RKIP (shRNA 501, 271,376) respectively. RKIP expression was analyzed by Western Blot. shRNA 271 caused significant down-regulation of RKIP expression. **d** In SW1116/RKIP^—^ cells, Western Blot verified the decrease in RKIP expression. **e** LoVo control: LoVo cells transfected with empty pIRES2-EGFP; PEBP-1-a: LoVo cells transfected with over-expression vector pIRES2-EGFP/PEBP-1, PEBP-1-b is repeated sample to PEBP-1-a. **a**, **b**, Magnification ×400). *Abbreviations: RKIP,* Raf kinase inhibitor protein; *NCRCT,* non neoplastic colorectal tissue; *CRCT,* colorectal cancer tissue; *MLNT,* metastatic lymph node tissue; *PEBP,* Phosphatidylethanolamine-binding proteins

SW1116 with the highest RKIP protein in several cell lines were transfected with RKIP shRNA by lipofectamine and observed by fluorescence microscopy. The results showed a high transfection efficiency, while the plasmid shRNA271 had an obvious interfering effect (Fig. 4c). Positive clones were screened by G418 (Fig. 4a) and identified by the green fluorescent protein (GFP) expression (Fig. 4b), and named SW1116/RKIP^**—**^ cells. The best screening concentration of G418 for SW1116 cells was 800ug/mL. The results showed the single cell clone all expression GFP (Fig. 4b). The RKIP protein expression was lower in SW1116/RKIP^—^ than in SW1116 cells (Fig. 4d). In the poorly differentiated LoVo cells, that were transfected with the eukaryotic expression vector pIRES2-EGFP/PEBP-1 plasmid, an up-regulation of RKIP expression was observed (Fig. 4e).

### Down-regulation of RKIP promotes invasion in SW1116 cells while its up-regulation inhibits invasion and migration in LoVo cells

To investigate the role of RKIP in human colorectal cancer invasion and metastasis, matrigel invasion assay was used to detect the difference of invasion and metastatic ability in SW1116 cells, SW1116/ RKIP^**—**^ cells, LoVo cells and LoVo cells transfected with pIRES_2_-EGFP/PEBP-1.

**Fig. 5:**
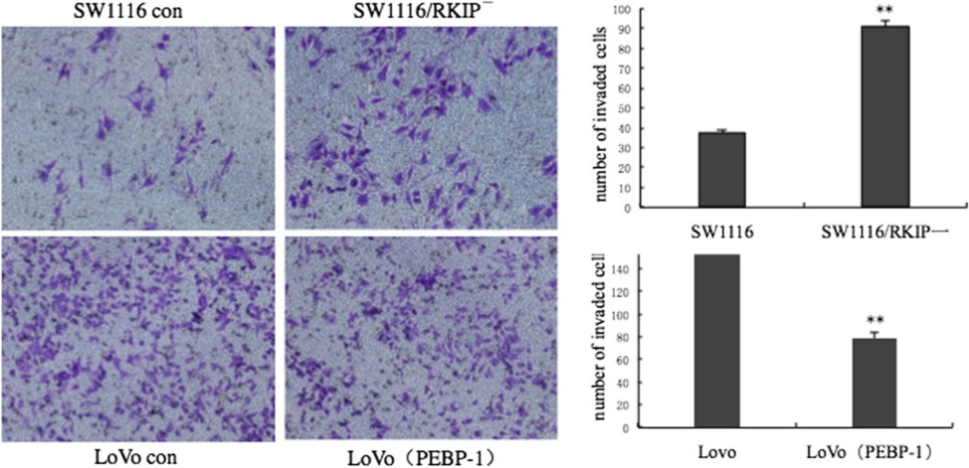
The effect of RKIP gene on invasiveness of cells: Significantly higher number of invasive cells in SW1116 /RKIP^**−**^ cells with RKIP down-regulation, as compared to SW1116 control cells (*P* <0.05); Decreased number of invasive cells in LoVo PEBP-1 compared to LoVo con (*P* <0.05). *Abbreviations: RKIP,* Raf kinase inhibitor protein*; LoVo PEBP-1,* LoVo cells transfected with over-expression vector pIRES_**2**_-EGFP/PEBP-1; *LoVo con*, LoVo control cells

Under the same culture conditions and inoculation density, the down-regulation of RKIP in SW1116/RKIP^**—**^ increased the number of migrated cells on the lower surface of the matrigel-coated transwell membrane (90.67 ± 4.04 *vs*. 37.33 ± 2.51, *P* <0.01) at 72 h. The up-regulation of RKIP in LoVo cells transfected with pIRES_2_-EGFP/PEBP-1 reduced the number of migrated cells (79.24 ± 5.18 *vs*. 154.33 ± 7.25, *P* <0.05) at 72 h (Fig. 5). There were no cell across the membrane at 24 h, and only few cells were found across it in both the groups at 48 h.

### RKIP regulates cell cycle and the down-regulation of RKIP inhibits G1 cell cycle arrest and promotes cell proliferation: role of RKIP in cell cycle regulation

**Fig. 6:**
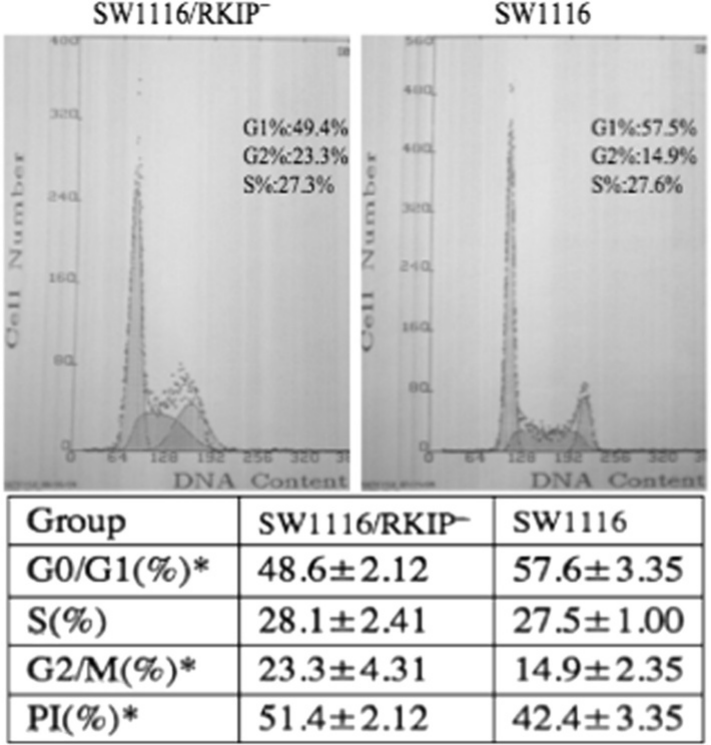
The role of RKIP gene on cell cycle arrest and cell proliferation; cell cycle arrest and cell proliferation index as analyzed by flow cytometry: Down-regulation of RKIP inhibited G1 cell cycle arrest and promoted cell proliferation. G0/G1 %, G2/M % and PI % are significantly higher in SW1116/RKIP^**—**^ cells with RKIP down-regulation, as compared to control SW1116 cells; *Abbreviations: RKIP,* Raf kinase inhibitor protein*; PI %,* Proliferation index

To avoid the impact of transfection reagent on cell cycle, we selected SW1116/RKIP^**—**^ cells with steady down-regulation of RKIP expression, instead of SW1116 cells transfected with RKIP shRNA. The percentage of cells at S and G2/M phases by FCM was analysed by calculating the PI. PI % = (S + G2/M)/(G0/G1 + S + G2/M)×100 was calculated. Down-regulation of RKIP decreased the cells in G0/G1 phase (48.6 ± 2.12 *vs.* 57.6 ± 3.35, *P* <0.05) and increased the cells in G2/M phase (23.3 ± 4.31 *vs*. 14.9 ± 2.35, *P* <0.05), thereby increasing the PI (51.4 ± 2.12 *vs*. 42.4 ± 3.35, *P* <0.05) (Fig. 6). This suggests an important effect of RKIP on the invasion and migration of SW1116 and LoVo cells, and that down-regulation of RKIP inhibits G1 cell cycle arrest and promotes cell proliferation. At the same time, LoVo cells transfected with pIRES_2_-EGFP/PEBP-1 and LoVo cells transfected with pIRES2-EGFP negative control plasmid were analyzed through flow cytometry to observe the effect of over-expression of RKIP gene on the ability of cell cycle. There were no significant difference between the two groups.

### Down-regulation of RKIP inhibited chemosensitivity of CRC cells to Hydroxycamptothecine

**Fig. 7:**
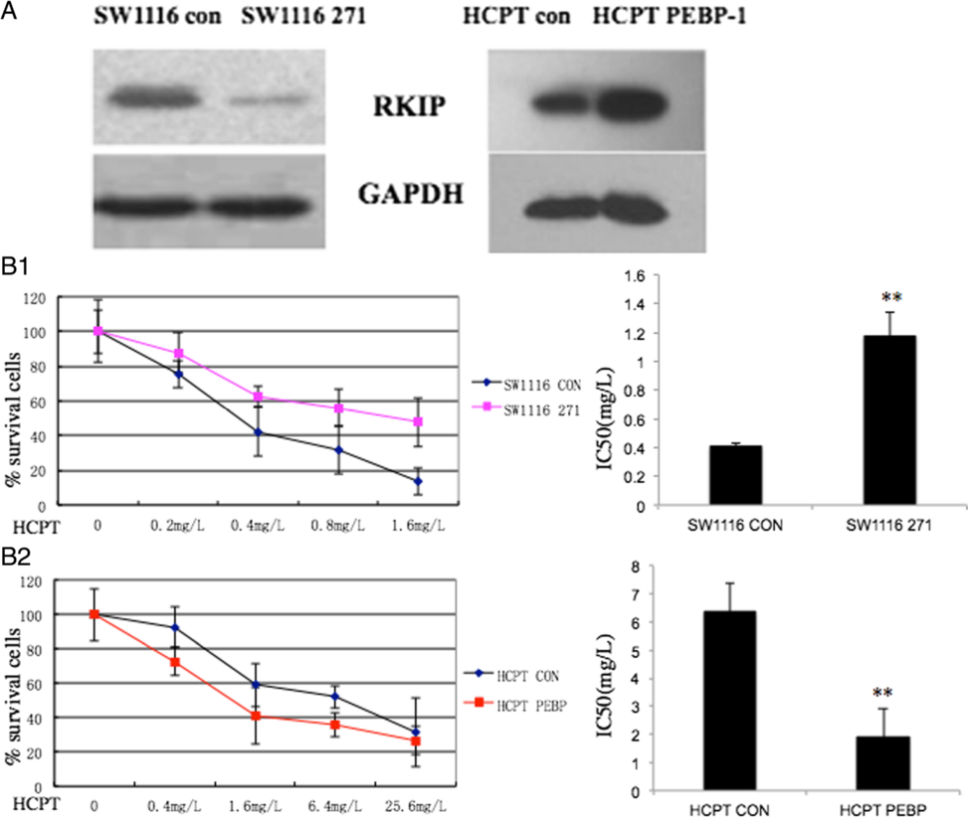
Effect of RKIP on sensitivity of CRC cells to hydroxycamptothecine (HCPT); **a** RKIP expression in each cell group. SW1116 con: SW1116 cells transferred with empty pIRES2-EGFP vector; HCPT con: SW1116/HCPT cells transferred with empty pIRES2-EGFP vector; SW1116 271: SW1116 cells transferred with shRNA vector targeted RKIP(PGPU6/GFP/Neo-PEBP1-271); HCPT PEBP-1: SW1116/HCPT cells transferred with over-expression pIRES2-EGFP/PEBP-1 vector; **b** 5 × 10^3^ cells incubated in 96-well culture plate for 24 h followed by treatment with different concentrations of HCPT for 48 h. Cell survival rate determined by MTT assay. IC50 values of HCPT analyzed by SPSS software. *Abbreviations: RKIP,* Raf kinase inhibitor protein, *HCPT*, hydroxycamptothecine; MTT: (3-(4,5-dimethylthiazol-2-yl)-2-5 diphenyl tetrazolium bromide) assay

The effects of RKIP expression on the chemosensitiviy of CRC to HCPT were observed by MTT (Fig. 7). The results represent mean ± SD of three experiments. Hydroxycamptothecin-resistant cell line (SW1116/HCPT), derived from human colon cancer cell line SW1116 by treatment with step-wise increasing concentrations of HCPT has been described previously [22]. RKIP protein expression levels were verified again (Fig. 7a). Down-regulation of RKIP increased IC50 values of HCPT in SW1116 cells: 1.176 mg/L (SW1116 con) *vs*. 0.410 mg/L (SW1116 271) (Fig. 7b1); whereas up-regulation of RKIP decreased the IC50 values of HCPT in SW1116/HCPT cells: 1.891 mg/L (HCPT PEBP) *vs*. 6.374 mg/L (HCPT CON) (Fig. 7b2). The results show that RKIP increases chemosensitivity of CRC cells to HCPT.

### Down-regulation of RKIP inhibits HCPT-induced apoptosis

**Fig. 8:**
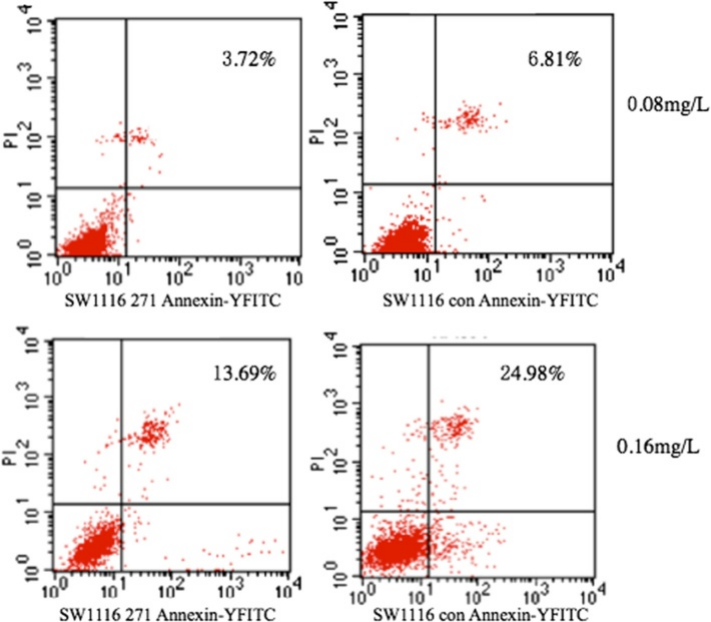
Apoptosis induction by hydroxycamptothecine (HCPT) in two group cells. Each group was treated with HCPT for 48 h at 0.08 mg/L, 0.16 mg/L concentrations. 1 × 10^6^ cells were collected and analyzed by flow cytometry after staining with Annexin V/PI

After treatment with HCPT, SW1116 cells transfected with shRNA 271 vector and control vector for 48 h were analysed for evidence of apoptosis. As previously demonstrated, shRNA 271 vector down-regulate the expression of RKIP. The apoptosis rate was lower in SW1116 cells transfected with shRNA 271 vector than that in control cells (0.08 mg/mL: 6.91 % ± 1.03 % *vs*. 3.35 % ± 1.12 %; 0.16 mg/mL; 22.54 % ± 2.24 *vs*. 13.80 % ± 1.61, *P* = 0.019 and *P* = 0.005, respectively; Fig. 8). It can be inferred from these experiments that that the down-regulation of RKIP inhibits the chemosensitivity of CRC cells to HCPT by inhibiting apoptosis.

## Discussion

Ras kinase inhibitor protein (RKIP) has been shown to be associated with metastatic disease in an increasing number of solid tumors. Dysregulation of RKIP expression can potentially be associated with many malignancies. RKIP is initially expressed at a lower level in human metastatic prostate cancer cells as compared to that in non-metastatic cancer cells, and may have a role in preventing vascular invasion of these cells [23]. RKIP expression is significantly decreased in insulinomas [24], anaplastic thyroid tumors [25], cutaneous squamous cell carcinoma [26], endometrial cancer [27], and hepatocellular carcinoma [28]. Studies have also shown reduced RKIP expression in lymph node metastases of breast and melanoma cancer [29, 8].

Several studies have demonstrated an association of RKIP with clinicopathological features in colorectal cancer. RKIP is depleted in distant metastases of both mismatch repair proficient and deficient colorectal cancers [18], and is a prognostic marker for disease-free survival [20] and distant metastatic disease [30]. Loss of RKIP in tumour buds has been frequently reported, with 60.8 % of cases sustaining a complete loss in this area of CRC. Moreover, the loss of RKIP can help predict the dynamics of epithelial-mesenchymal transition (EMT) [31]. In our study the RKIP expression was decreased in CRCT when compared with NCRCT; but probably not to such an extent.

To the best of our knowledge, no *in vivo* and *in vitro* studies focusing on RKIP function in colorectal cancer cells have been conducted. Immunostaining results obtained in our study highlighted that RKIP is depleted in lymph node metastases and the activation of ERK is significant in CRC, both in-situ as well as in the CRC cell lines and metastatic lymph node tissue. There was a negative correlation between expression of RKIP and P-ERK. Moreover, our study demonstrated that RKIP expression was associated with the degree of differentiation of colorectal cancer cells, with higher expression occurring in well-differentiated cell lines (HT-29, SW1116) than in poorly differentiated cell lines (LoVo). Similarly, other studies have also demonstrated that Raf/MEK/ERK signaling pathway is associated with not only metastatic disease, but also differentiation in certain cell lines [32]. RKIP can induce differentiation and repression of cell proliferation in keratinocytes [33]. RKIP contributes to the monocytic differentiation process via inhibition of the NFkB signaling cascade, which is independent from the canonical Ras/Raf/MEK/ERK pathway [34]. Furthermore, RKIP has been shown to enhance neuronal differentiation via enhanced crosstalk from PKC to ERK-1/2, and enhancement of G-protein-coupled receptor signaling [35]. Some studies have suggested that RKIP may be dissociated from Raf-1 after phosphorylation at serine 153 by PKC. In effect, PKC appears to relieve a key inhibitor of the Raf/MAP kinase signaling cascade [36]. As time constraints did not allow for a differential gene expression analysis, the details of underlying signaling pathway could not be determined.

Poorly differentiated LoVo cells transiently transfected with pIRES2-EGFP/PEBP-1 plasmid to cause over-expression of RKIP, were tested in matrixgel invasion and cell cycle assay. Over-expression of RKIP was found to inhibit invasion, but did not affect cell cycle and PI of LoVo cells. However, loss of RKIP function was found to inhibit G1 cell cycle arrest and increase cell PI (PI %) in SW1116 cells (Fig. 6, PI: 51.4 ± 2.12 vs. 42.4 ± 3.35) in our study, the difference of PI is less than one fold; While the down-regulation of RKIP in SW1116/RKIP^**—**^ increased the number of migrated cells on the lower surface of the matrigel-coated transwell membrane (Fig. 5, 90.67 ± 4.04 *vs*. 37.33 ± 2.51, *P* <0.01), the difference is two to three folds. The cell proliferation could complicate the interpretation of the results probably, but we thought that the role of cell invasion was more significant than the cell proliferation in results concluded from Fig. 5 in our study.

SW1116/RKIP^**—**^ cells with stable down-regulation of RKIP expression were developed in our study, which perhaps minimized experimental error and cytotoxicity, as seen with the use of commercial liposome-based agents. Lipofectamine 2000, a cationic liposome based reagent, can change expression of marker genes for cell cycle inhibition or progression, such as p21 and PCNA, and also shows decrease in viability and DNA content [37]. We found that stable transfection as compared to transient transfection was more advantageous in terms of cell cycle assay.

A natural indole alkaloid extracted from a Chinese tree *Camptotheca cuminata*, HCPT is a topoisomerase I-specific inhibitor [38]. Available evidence shows that HCPT can induce apoptosis in multiple cancers [39], and can also inhibit metastatic colorectal cancer [40]. HCPT treatment activates caspase 3, and down regulates the expression of surviving [41]. Multidrug resistance gene ABCG2 and cell cycle related gene p21 had high expression in SW1116/HCPT cells [42, 22]. In our study, RKIP promoted cell apoptosis induced by HCPT, while the down-regulation of RKIP expression inhibited cell cycle arrest. Cell cycle capture allows cells to stop proliferating and repair the damage in order to continue cell division. Expression of p21 can protect cells from apoptosis induced by anticancer drugs. P21 is probably involved in RKIP regulated apoptosis induced by HCPT. RKIP overexpression appears to regulate tumor cell sensitivity to TRAIL by inhibiting YY1 and up-regulating DR5. RKIP overexpression in combination with TRAIL has been shown to potentiate the above effects and activate caspases 8, 9, and 3, resulting in apoptosis [43].

## Conclusions

In summary, RKIP is not only a metastasis inhibitor factor, but also related with colorectal cancer cell differentiation. Loss of RKIP inhibits cell cycle arrest and promotes cell proliferation. Furthermore, down-regulation of RKIP reduced chemosensitivity by inhibiting apoptosis induced by HCPT. Our findings suggest that further investigation into the pathophysiological mechanisms of RKIP may help develop new therapeutic strategies for the treatment of colorectal cancer. Moreover, RKIP may serve as an immune surveillance cancer gene since its low expression (or absence) may serve as a marker for a likely poor immune response, that could serve to shield the tumor cells against the host cytotoxic effector cells.
